# In Silico Evaluation of the Potential Association of the Pathogenic Mutations of Alpha Synuclein Protein with Induction of Synucleinopathies

**DOI:** 10.3390/diseases11030115

**Published:** 2023-09-06

**Authors:** Mohamed E. Elnageeb, Imadeldin Elfaki, Khalid M. Adam, Elsadig Mohamed Ahmed, Elkhalifa M. Elkhalifa, Hytham A. Abuagla, Abubakr Ali Elamin Mohamed Ahmed, Elshazali Widaa Ali, Elmoiz Idris Eltieb, Ali M. Edris

**Affiliations:** 1 Department of Basic Sciences, College of Applied Medical Sciences, University of Bisha, P.O. Box 551, Bisha 61922, Saudi Arabia; 2 Department of Biochemistry, Faculty of Science, University of Tabuk, P.O. Box 741, Tabuk 71491, Saudi Arabia; 3 Department of Medical Laboratory Sciences, College of Applied Medical Sciences, University of Bisha, P.O. Box 551, Bisha 61922, Saudi Arabia; 4 Department of Clinical Chemistry, Faculty of Medical Laboratory Sciences, University of El Imam El Mahdi, Kosti 27711, Sudan; 5 Department of Anatomy, Faculty of Medicine and Health Sciences, Nile Valley University, Atbara 46611, Sudan

**Keywords:** synucleinopathies, Parkinson’s disease (PD), alpha-synuclein (α-Syn) protein, SNCA gene non-synonymous single nucleotide polymorphisms (nsSNPs), bioinformatics, synucleinopathies

## Abstract

Alpha synuclein (α-Syn) is a neuronal protein encoded by the SNCA gene and is involved in the development of Parkinson’s disease (PD). The objective of this study was to examine in silico the functional implications of non-synonymous single nucleotide polymorphisms (nsSNPs) in the SNCA gene. We used a range of computational algorithms such as sequence conservation, structural analysis, physicochemical properties, and machine learning. The sequence of the SNCA gene was analyzed, resulting in the mapping of 42,272 SNPs that are classified into different functional categories. A total of 177 nsSNPs were identified within the coding region; there were 20 variants that may influence the α-Syn protein structure and function. This identification was made by employing different analytical tools including SIFT, PolyPhen2, Mut-pred, SNAP2, PANTHER, PhD-SNP, SNP&Go, MUpro, Cosurf, I-Mut, and HOPE. Three mutations, V82A, K80E, and E46K, were selected for further examinations due to their spatial positioning within the α-Syn as determined by PyMol. Results indicated that these mutations may affect the stability and function of α-Syn. Then, a molecular dynamics simulation was conducted for the SNCA wildtype and the four mutant variants (p.A18G, p.V82A, p.K80E, and p.E46K). The simulation examined temperature, pressure, density, root-mean-square deviation (RMSD), root-mean-square fluctuation (RMSF), solvent-accessible surface area (SASA), and radius of gyration (Rg). The data indicate that the mutations p.V82A, p.K80E, and p.E46K reduce the stability and functionality of α-Syn. These findings highlight the importance of understanding the impact of nsSNPs on α-syn structure and function. Our results required verifications in further protein functional and case–control studies. After being verified these findings can be used in genetic testing for the early diagnosis of PD, the evaluation of the risk factors, and therapeutic approaches.

## 1. Introduction

Parkinson’s disease (PD) is a neurodegenerative disorder associated with motor deficit and characterized by the gradual degeneration and loss of dopaminergic neurons located in the pars compacta of the substantia nigra region of the brain [1]. This is accompanied by decreased levels of dopamine in the striatum [1]. It affects more than 1% of the population over the age of 60 years old [2]. It is the second most common neurodegenerative disease after Alzheimer’s disease and its prevalence is expected to be doubled by the year 2030 [2]. PD can be identified by a range of motor symptoms, including rest tremors, rigidity, bradykinesia, and loss of postural reflexes [3,4]. Apart from the motor symptoms, Parkinson’s disease is distinguished by a variety of non-motor symptoms, including cognitive impairment, depression, autonomic dysfunction, sleep dysfunction, and anxiety [2,3]. Alpha-synuclein (α-Syn) is an acidic protein with a small molecular weight of 14 KDa found in neurons of the central and peripheral nervous system, blood, and other cells [5]. α-Syn is composed of an N-terminus (amino residues 8 to 32) and different beta pleated sheets found between the amino acid residues 35 to 56, followed by the amino acid residues 61 to 95 (NAC region). The C-terminus appears in an unstructured region [6]. PD is characterized by intraneuronal accumulation of inclusions of misfolded and aggregated α-synuclein [7]. These inclusions are called Lewy bodies (LBs) and Lewy neurites [7]. These inclusions can spread from cell to cell and to different part of the brain tissues [3,7]. It has been reported that that aggregates of alpha-synuclein (α-Syn) exert toxic effects on the neuron, resulting in neuronal degeneration via mechanisms, such as defective mitochondria, dysfunction of lysosomes, and cells membrane [8,9,10], in addition to the stress of endoplasmic reticulum and synaptic abnormality [8,9,10]. The pathological aggregation of α-Syn is a common lesions neurodegenerative disorders called the synucleinopathies for example Parkinson’s disease, dementia with Lewy bodies (DLB), and multiple system atrophy (MSA) [11]. The function, binding partners, or post-translational modifications of α-Syn remain to be determined and the precise native structure of α-Syn is not well solved. It was reported as disordered, alpha helical, or a mixture of disordered and helical [12]. Under physiologic conditions, native α-Syn remains in balance between unfolded monomers and alpha helically folded tetramers with a reduced tendency to aggregate [13,14]. The decreased ratio of tetramer to monomer leads to elevated levels of α-Syn unfolded monomers [13,14]. This unfolded monomeric form tends to form aggregates [13,14]. The aggregation of α-Syn leads a conformational change of the α-Syn as it becomes rich in beta pleated sheet that enable the aggregates to form oligomeric α-Syn, protofibrils, and then fibrils which form the Lewy bodies [9]. SNCA gene variations and point mutations were reported to be risk factor for PD [9,15,16]. For example, whole-locus triplications and the mutations H50Q, A30P, E46K, A53T, and G51D [17,18]. In the present study we have investigated the association of non-synonymous single nucleotide polymorphisms (nsSNPs) in SNCA gene with Parkinson’s disease using bioinformatics tools.

## 2. Methodology

### 2.1. Plane of Work

**Figure 1:**
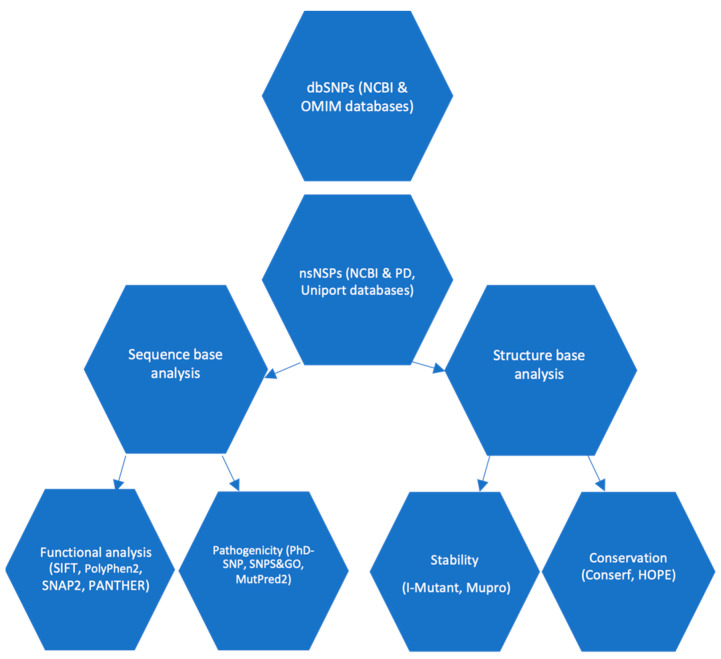
Plan of work.

The current research utilized a computational approach that was in line with previous studies [19,20,21]. The study entailed the utilization of diverse bioinformatics tools to predict the harmful impacts of nsSNPs present in the SNCA gene and α-syn protein. Subsequently, nsSNPs exhibiting a high-risk profile were selected for further analysis, entailing an assessment of their degree of conservation, stability, and structural influence. The methodology utilized for the identification and categorization of potential functional nsSNPs in the SNCA gene is illustrated in Figure 1.

The plan used for the identification of potential non-synonymous single nucleotide polymorphisms in the SNCA gene that may affect α-syn protein structure and function.

### 2.2. Data Collection

The nucleotide and amino acid sequences of the α-syn protein, identified by accession numbers NG_011851.1 and NP_000336.1, respectively, were procured from the NCBI database in FASTA format. The NCBI database is accessible via the following web address: http://www.ncbi.nlm.nih.gov (accessed on 23 May 2023). The SNP data for the SNCA gene is available on the SNP database of the NCBI and can be accessed via the following URL: http://www.ncbi.nlm.nih.gov/snp/ accessed on 23 May 2023. Furthermore, pertinent data regarding the SNCA gene and protein was procured from the PDB database, Uniprot database (https://www.uniprot.org/uniprotkb/P37840/entry accessed on 23 May 2023) and Online Mendelian Inheritance in Man (OMIM) database, which can be accessed at http://www.omim.org (accessed on 23 May 2023). 

### 2.3. Exploring the Influence of SNPs on Protein Function

In this section, we explore the predictions regarding the influence of genetic variation on protein function. Various online tools and servers, such as SIFT Sequence (https://sift.bii.a-star.edu.sg/www/SIFT_seq_submit2.html accessed on 23 May 2023) [22], polyPhen2 (http://genetics.bwh.harvard.edu/pph2 accessed on 23 May 2023) [23], SNAP2 (https://www.rostlab.org/servces/snap/ accessed on 23 May 2023) [24], and PANTHER (http://www.pantherdb.org/tools/csnpScoreForm.jsp accessed on 23 May 2023) [25] were utilized to evaluate the potential effects of SNPs. The aforementioned tools provided distinct methodologies for the classification of nsSNPs and the determination of confidence scores based on a variety of input criteria. The functional impact of non-synonymous single amino acid substitutions was determined with the aid of SIFT, PolyPhen2, SNAP2, and PANTHER predictions. These tools were used to distinguish between tolerable and damaging effects on protein function. The SIFT method used a tolerance index score to evaluate genetic variations, classifying those with scores lower than 0.05 as deleterious. In contrast, the PolyPhen algorithm allocated a numerical value between 0 and 1, where 0 signified a neutral impact and 1 denoted the most deleterious impact. In contrast, SNAP2 conducted a comparative analysis of genomes and made predictions regarding the potential functional consequences at the amino acid level. Finally, the PANTHER tool utilized a scoring mechanism ranging from −1 to 1 to assess the anticipated influence of the input SNPs on the functionality of proteins. The functional impact of the score was indicated by its sign in PANTHER, where positive scores were indicative of functional impact and negative scores were suggestive of non-functionality. Greater absolute values indicated a more robustly anticipated effect on protein functionality. The SNPs present in the SNCA gene that were found to be deleterious common across all four servers were subjected to further computational analysis.

### 2.4. Prediction of SNP-Disease Associations

The tools employed for evaluating the correlation between the filtered SNPs and the disease were PhD-SNP [26] and SNPS&GO [27], accessible at http://snps.biofold.org/phd-snp/phd-snp.html (accessed on 6 July 2023) and http://snps-and-go.biocomp.unibo.it/snps-and-go/ (accessed on 6 July 2023), respectively. The SNPs&GO webserver is a support vector machine (SVM) that utilizes computational methods to detect harmful single amino acid substitutions. This tool is based on scientific principles and is designed to assist in the identification of such substitutions. The classifier that is based on SVM is comprised of a solitary SVM that receives as input the protein sequence, profile, and functional information. The classification of a missense variant into disease-related or neutral is accomplished through the utilization of Gene Ontology (GO) annotations. The input parameters for this process include the amino acid sequence or Swiss-Prot code, GO terms, and amino acid substitutions. When the SNPs&GO score surpasses 0.5, it is indicative of a mutation that causes a disease.

The Predictor of human Deleterious Single Nucleotide Polymorphisms (PhD-SNP) employs a classifier based on SVM to categorize variants associated with diseases. The utilization of sequence and profile data is integral to the categorization of amino acid substitutions as either neutral or disease-associated during the classification procedure. The calculation of the sequence profile involves the utilization of an input vector that is obtained from the frequencies of amino acids in both the wildtype and mutant sequences, the number of aligned sequences, and the conservation score at the substituted location. When the PhD-SNP score surpasses 0.5, it suggests the presence of a mutation that is responsible for causing a disease.

### 2.5. Predicting the Impact of SNPs on Protein Stability

The I-Mutant algorithm utilizes SVM methodology in order to predict alterations in protein stability resulting from missense nsSNP. The output of this particular tool is a reliability index (RI) that spans from 0 to 10, where the maximum value of 10 denotes the utmost degree of reliability. The I-Mutant (http://folding.biofold.org/i-mutant/i-mutant2.0.html accessed on 6 July 2023) algorithm was provided with the amino acid sequence of the α-Syn protein, along with information regarding the specific residues that had undergone mutations, including their respective positions. The MUpro tool, which can be accessed at https://mupro.proteomics.ics.uci.edu, (accessed on 6 July 2023) was employed to evaluate the stability. MUpro is a collection of machine learning algorithms that have been designed to forecast the impact of individual amino acid mutations on protein stability. The utilization of two machine learning techniques, specifically SVM and Neural Networks, is observed in the web server tool. The aforementioned techniques underwent training using a comprehensive dataset of mutations. The effectiveness of both methodologies was evaluated using a 20-fold cross-validation technique, resulting in an accuracy rate exceeding 84% and calculated a score that ranged from −1 to 1 as the prediction reliability. One significant advantage of the employed methodologies is their capacity to predict modifications in protein stability, irrespective of tertiary structures.

### 2.6. Prediction of SNPs on α-Syn Protein Functionality in Relation to Structure Using Mut-Pred Server

The MutPred2 (http://mutpred.mutdb.org) web server (accessed on 7 July 2023) is used for the classification of a mutation as either neutral or disease-associated [28]. The utilization of a machine-learning-based technique is employed to estimate the molecular mechanism of pathogenicity of an amino acid substitution. The aforementioned tool utilizes a comprehensive set of fifty distinct protein properties in order to determine the impact of substitutions. A MutPred2 score exceeding 0.5 is indicative of a mutation with pathogenic potential.

### 2.7. Analyzing Protein Sequence Conservation Using ConSurf

The analysis of amino acid conservation is conducted through the utilization of the ConSurf web server, which can be accessed at https://consurf.tau.ac.il accessed on 23 May 2023. Through the evaluation of conservation levels, this algorithm is capable of discerning the functional regions present within a given protein. The conservation grades, which are assigned values between 1 and 9, serve as an indicator of the degree of evolutionary conservation exhibited by individual amino acids. According to Ashkenazy et al., 2010 [29], the conservation level is rated on a scale of 1 to 9, where a grade of 9 represents the highest conservation and a grade of 1 represents the lowest conservation.

### 2.8. Analysis of Properties of Proteins

The HOPE software tool has been developed with the purpose of automating the analysis of protein mutants. The resource in question is available for access via the following URL: https://www.cmbi.umcn.nl/hope accessed on 23 May 2023. The HOPE tool employs data from the UniProt database to evaluate the effects of single nucleotide variations on the structural and functional properties of proteins. The aforementioned tool produces a detailed report that encompasses written explanations, graphical illustrations, and dynamic visualizations, thereby presenting a comprehensive depiction of the impact of the mutation (Venselaar et al., 2010) [30].

### 2.9. Determine the Active Binding Site Using PyMol Software

The software PyMol Version 2.0 was employed to identify the specific binding site where modifications in the amino acid composition occurred. The analysis of residues situated within the active site is a key aspect of employing pyMOL. The software mentioned above is an open-source tool used for molecular visualization. The technology possesses the capability to generate high-quality three-dimensional representations of small molecules. Therefore, three candidate mutants (V82A, K80E, E46K) were chosen from a pool of the top 20 nsSNPs identified as deleterious mutants. These mutants were subsequently subjected to MD simulation for further analysis.

### 2.10. Performing of Molecular Dynamics Simulations (MDS)

To examine the structural changes over time in both wildtype and mutant structures, MDS were performed using GROMACS version 2020.6 on a Google Collaboration pro notebook with high RAM. The GROMACS-OPLS-AA force field was employed for the initial calculation. The systems were immersed in a cubic box that was half full of water molecules with marginal radii of 1 nm. Using the GROMACS genion tool, 10 sodium Na+ ions were then added to the simulation box to neutralize the system. The steepest descent algorithm, with an energy step size of 0.01 and a maximum number of 50,000 iterations, was used to complete the energy minimization. A Parrinello–Rahman (pcouple) pressure of 1 bar and a Berendsen temperature (tcouple) of 300 K were used to keep the system stable. For temperature and pressure, the coupling constant was changed to 0.1 and 2.0 ps, respectively. With the help of the partial mesh Ewald (PME) technique, the electrostatic interactions in the system were calculated. The short-range cut off for electrostatic (rcoulomb) and van der Waals (rvdw) interactions was established at 1.0 nm. Every 10 ps, the neighbor list (nstlist) is updated. The LINCS algorithm, with a time step of 0.002 ps preserved all bond restrictions, including heavy atom-H bonds. It was decided to use an isothermal compressibility of 4.5 × 10^−5^. The coupling constants for temperature and pressure were set to 0.1 ps and 2.0 ps, respectively, and the system was equilibrated for 100 ps in each of the NPT (constant number of particles, pressure, and temperature) and NVT (constant number of particles, volume, and temperature) ensembles with Berendsen temperature (tcouple) at 300 K and Parrinello–Rahman (pcouple) pressure at 1 bar. Following that, trajectories were recorded every 1 ps during 10 ns of molecular dynamics simulations for both the natural and mutant structures. Various tools, including g_rms, g_rmsf, g_sasa, g_Rg, and g_density, were used to analyze RMSD (root-mean-square deviation), RMSF (root-mean-square fluctuation), SASA (solvent accessible surface area), Rg (radius of gyration), temperature, pressure, and density plots in order to compare structural deviations between the wildtype and mutant structures. The application XMGRACE was used to create each plot.

## 3. Results

The complete set of SNPs associated with the SNCA gene and its corresponding protein sequence (identified by Uniprot ID P37840) were obtained from the NCBI dbSNP and Uniprot Knowledgebase databases, respectively. The SNCA gene sequence was mapped for a total of 42,272 SNPs belonging to various functional classes, as illustrated in Figure 1. Among the 42,272 SNPs identified, a total of 177 nsSNPs were detected in the coding region. These nsSNPs have the potential to cause missense or nonsense mutations, which can significantly impact the structure and function of the protein. The study considered the nsSNPs present in the coding sequence of the α-syn protein.

### 3.1. Predicting nsSNP Deleterious on α-Syn Protein

Our research focused on the analysis of nsSNPs to determine their potential impact on the structural and functional properties of the α-syn protein. Out of the 177 nsSNPs, only 20 were deemed entirely deleterious or with a high probability of being so by SIFT and PolyPhen, as presented in Table 1. The SIFT filtration process yielded score values of 0, whereas the PolyPhen filtration process yielded values ranging from 0.08 to 1. PolyPhen identified four nsSNPs (V82A, K80E, E46K, and A18G) as potentially harmful. Moreover, confirmation prediction by SNAP2 and PANTHER have corroborated their harmful effects (Table 1). A total of 20 nsSNPs were identified as the most deleterious based on prediction software.

### 3.2. Analysis of Disease-Associated nsSNPs

The present study employed a combined approach utilizing PhD-SNP and SNP&GO tools to investigate a set of 20 nsSNPs in order to obtain more profound insights into their disease susceptibility. Analysis utilizing PhD-SNP methodology has successfully identified four nsSNPs that demonstrated a significant correlation with the disease in question. Conversely, the remaining 16 nsSNPs were predicted to have neutral effects. The results of the SNP&GO analysis suggest that all 20 nsSNPs have the potential to be associated with disease. Subsequent examination of the SNCA gene has revealed the presence of four nsSNPs that have been categorized as variants associated with pathological conditions as shown in Table 2. The four variants (V82A, K80E, E46K, and A18G) in question were subjected to additional analysis in order to obtain a thorough comprehension of their potential impact, given their classification as the most harmful nsSNPs.

### 3.3. The Impact of Predicted Deleterious Mutations on α-Syn Protein Stability

An investigation was conducted utilizing the I-mutant 2.0 and MuPro software tools to evaluate the impact of four nsSNPs, specifically V82A, K80E, E46K, and A18G, on the stability of a protein. The findings derived from both I-mutant 2.0 and MuPro exhibited coherence, indicating that all four nsSNPs are anticipated to diminish the protein’s stability (Table 3).

### 3.4. Investigation the Molecular Mechanisms Underlying Pathogenicity

Table 4 displays the probability scores derived from the MutPred2 server analysis for four distinct nsSNPs. The MutPred analysis findings suggest that three mutants, namely V82E, K80E, and E46K, possess the capability to elicit alterations in the protein’s structure. The changes seen in the study are the lack of pyrrolidone carboxylic acid at residue Q79, the lack of methylation at residue K80, the lack of ubiquitylation at residue K80, the addition of methylation at residue E46, changes in the disordered interface, and changes to the transmembrane protein. The MutPred prediction for the A18G mutant indicates a neutral impact on protein functions (Table 4).

### 3.5. Analysis of the Phylogenetic Conservation of nsSNP

**Figure 2:**
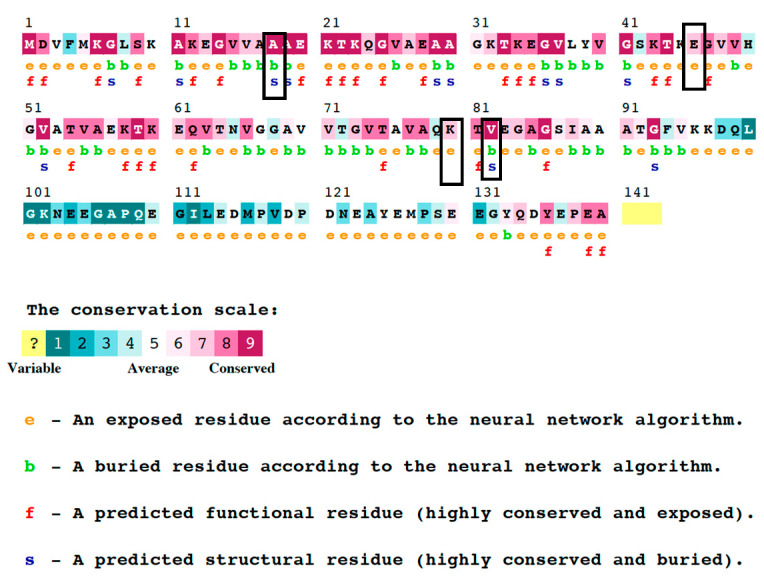
Outcomes of the ConSurf study in terms of residue conservation. The amount of confidence in the sequence conservation is shown by a range of colors in the ConSurf results. The sky-blue color in this color scheme stands in for variable residues, while the dark purple color stands in for highly conserved residues.

Table 5 presents the outcomes of the conservation analysis that were acquired from the ConSurf server for the four mutants. The results of the analysis demonstrate that the V82A and A18G mutants display a notable conservation score of nine, indicating that these residues are conserved and situated within concealed structural regions (Figure 2). In contrast, residues K80E and E46K were determined to be highly exposed, possessing a conservation score of seven. This suggests that these residues are located in less conserved and exposed regions of the protein.

### 3.6. HOPE Predications of the α-Syn Protein Properties

The present study employed the HOPE methodology to prognosticate the effects of four harmful nsSNPs located within the SNCA gene on diverse amino acid features such as hydrophobicity, charge, size, and spatial structural function. The study identified three mutant residues (V82A, K80E, and A18G) that exhibited a reduction in size relative to their wildtype counterparts. This reduction in size may result in a loss of interactions. Conversely, the E46K mutant was found to be larger, which could potentially lead to steric hindrance. Furthermore, according to HOPE, the incorporation of two mutations (E46K and K80E) results in the introduction of a residue with an opposing charge to that of the wildtype. Additionally, the hydrophobicity of the wildtype residue is greater than that of the A18G mutant residue. With respect to the protein’s architecture, it can be observed that the wildtype residue exhibits a preference for A-strand as its secondary structure. Conversely, the V80A mutant residue displays a preference for an alternative secondary structure. As a result, the local conformation of the protein will experience a slight destabilization. Moreover, it is noteworthy that the mutation E46K is situated in a sequence of amino acids that has been designated as a distinctive region in UniProt. The sequence comprises 4X11AA tandem repeats of (EGS)-K-T-K-(EQ)-(GQ)-V-X(4). The aforementioned mutation is situated in a sequence of amino acid residues that is reiterated within the protein, which is denoted as three, approximately. The substitution of an amino acid residue could potentially disrupt the structural integrity of this repetitive sequence, thereby compromising its functional properties.

### 3.7. Molecular Dynamic (MD) Simulation of α-Syn Protein and Its Mutants

#### 3.7.1. Temperature, Pressure, and Density

**Figure 3:**
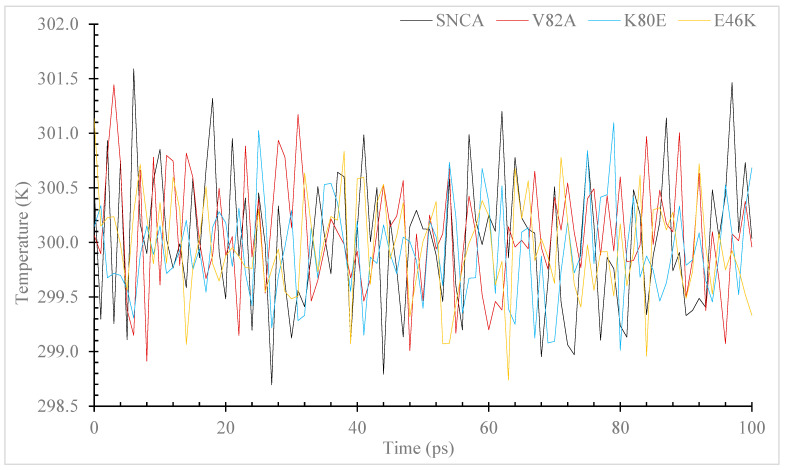
Simulation of the temperature changes over time for the α-syn protein wildtype and mutant versions. The simulation was performed using the GROMACS 5.1.2. The color black is used to symbolize the wildtype, whereas the colors red, blue, and yellow are used to represent the V82A mutant, K80E mutant, and yellow to represent the E46K mutant.

**Figure 4:**
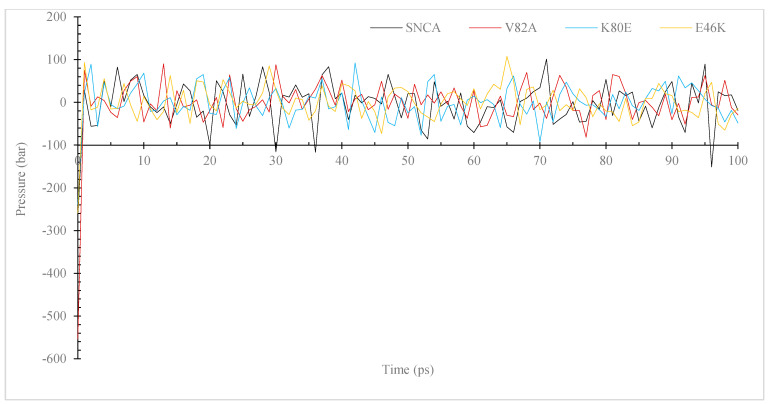
Simulation of the pressure changes over time for the α-Syn wildtype and mutant versions. The simulation was performed using the GROMACS program. The color black is used to symbolize the wildtype, whereas the colors red, blue, and yellow are used to represent the V82A mutant, K80E mutant, and yellow to represent the E46K mutant.

**Figure 5:**
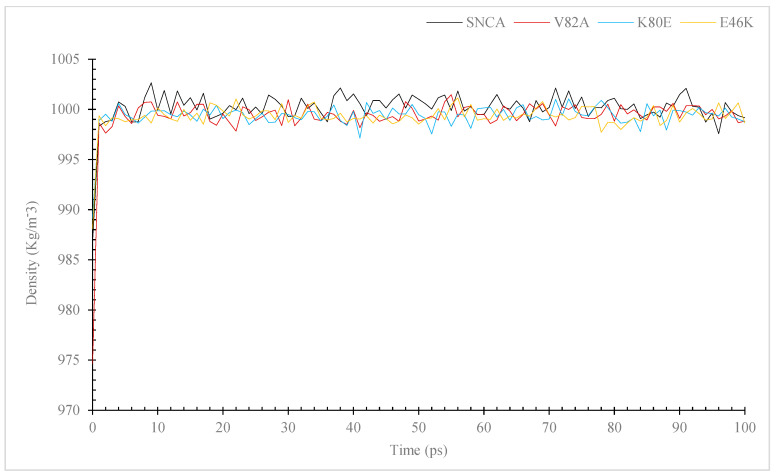
Simulation of the density changes over time for the α-syn protein wildtype and mutant versions. The simulation was performed using the GROMACS 5.1.2 program. The color black is used to symbolize the wildtype, whereas the colors red, blue, and yellow are used to represent the V82A mutant, K80E mutant and yellow to represent the E46K mutant.

During MD simulations, ions and solvents were balanced around the protein structures after energy minimization. NVT (thermostat) and NPT (bariostat) equilibrations were carried out to make sure that the molecules were oriented correctly within the structures. In an isothermal–isobaric ensemble, these two phases were used to stabilize the systems’ temperature and pressure, respectively. The NVT and NPT ensembles of the system were computed using the “gms/grompp” computational tool. Temperature, pressure, and density are all factors in the equilibrated process, which results in the creation of a density plot from the NPT. At a temperature of 300 Kelvin, Figure 3 shows the temperature profiles of the protein systems in both their natural and mutant forms. The plot shows how the conformations experienced large temperature swings throughout the 100 ps equilibrium phase. However, this behavior was not entirely unexpected because the majority of the conformations’ average temperatures were within a favorable range. For example, the native structure had an average temperature of 301.6 K, while the mutants V82A, K80E, and E46K had temperatures of 301.4, 301.1, and 301.1 K, respectively. The wildtype protein also displayed higher values. A density–time graph (Figure 4) and a pressure–time graph (Figure 5) were also produced using a time scale of 100 ps. The V82A, K80E, and E46K mutants revealed higher peaks of 90.3, 92.3, and 107.8 bars, respectively, than the wildtype structure, which had peaks above 101.3 bars, according to the pressure–time graph. While K80E (−238.6 bar) and E46K (−258.8 bar) mutants had greater values, the plot of pressure for mutant V82A shows a lower local minimum of −550 bar, which deviates from the natural structure’s value of −268.3 bars. According to the graph showing pressure changes over time, the wildtype structure’s mean pressure value across all conformations was −5.4 bars. However, the average pressure values for V82A, K80E, and E46K were, respectively, −2.6 bars, −2.2 bars, and −0.36 bars. The current work compares the wildtype α-syn protein’s density values to those of its mutant forms across a time scale of 100 ps (Figure 5). The wildtype protein and the mutant versions both have an average density value of 1000 kg/m^3^, with a few slight deviations. The mutant V82A, however, showed a reduced initial density value of 974.6 kg/m^3^, which is significant. All three mutants exhibit patterns of fluctuation similar to those of the native α-syn, but the mutants retain an average lower value of 999 Kg/m^3^.

#### 3.7.2. RMSD and RMSF

**Figure 6:**
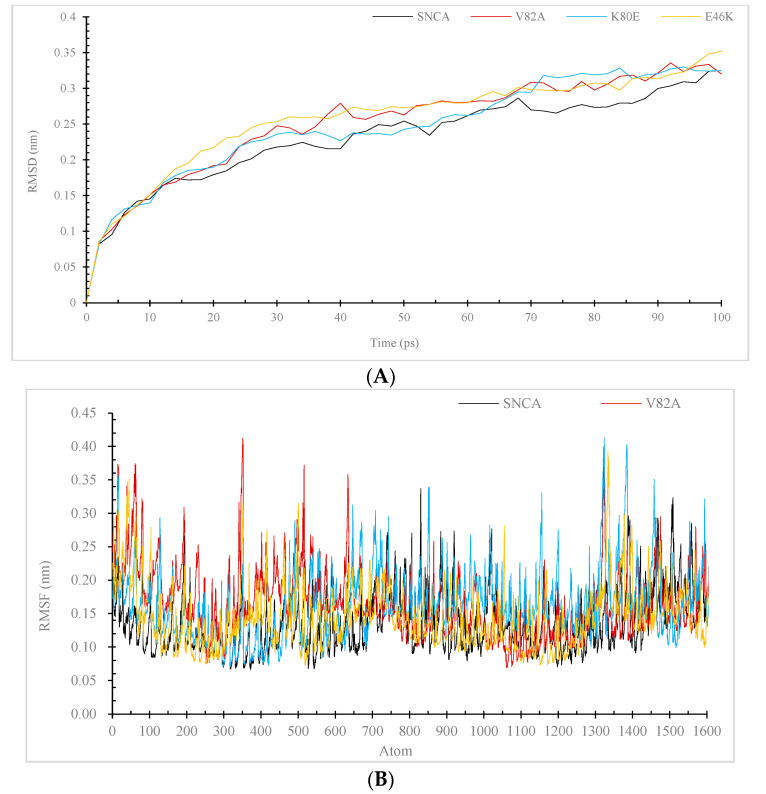
Difference between the root-mean-square deviation (RMSD) (**A**), and the root-mean-square fluctuation (RMSF) (**B**). For the backbone atoms of the α-Syn protein wildtype and mutant forms, graphs were created. The simulations were carried out with GROMACS 5.1.2. Black for to represent the wildtype, while red, blue, and yellow for the V82A, K80E, and E46K mutants, respectively.

This study utilized a molecular dynamics simulation methodology to contrast and depict the structural and functional dynamics of the SNCA gene’s wildtype and its three mutant forms, namely V82A, K80E, and E46K. The RMSD was utilized to analyze the trajectories of both native and mutant structures of the α-Syn. The RMSD graphs depicted in Figure 6 were produced by employing the trajectory file for the C-alpha backbone least square fit model via grms. The initial configurations of the three structures displayed a high degree of proximity. However, a sudden deviation was detected at 15 picoseconds, which led to an RMSD value of 0.18 nanometers for the three mutants. Subsequently, a gradual rise in RMSF measurements was observed in relation to the wildtype α-Syn.

The RMSD values of the wildtype and mutant structures were analyzed over a period of time (E46K 6A). The wildtype structure exhibited its highest RMSD value at 16 ps, whereas the V82A, K80E, and E46K mutants attained their maximum RMSD values at 14 ps, 15 ps, and 12 ps, respectively. The RMSD graph, which was generated by aligning the backbone of the wildtype α-syn with its V82A, K80E, and E46K mutants, demonstrated that the wildtype α-Syn displayed higher stability compared to its mutant counterparts. Following that, the previously mentioned mutants have been noted to affect the protein dynamics of SNCA in its natural state. The modifications observed in the RMSD plot of the mutants’ backbone serve as a suitable foundation for further examination. The examination of the RMSF values pertaining to both native and mutant residues was carried out in order to determine the effects of mutations on the atomic-level dynamic behavior, as depicted in Figure 6B. The RMSF of the native residues exhibited a range of 0.067 nm to 0.34 nm, with an average value of 0.137 nm. The RMSF values of the V82A, K80E, and E46K residues were computed to vary between 0.069 to 0.423, 0.071 to 0.413, and 0.071 to 0.392, respectively, exhibiting mean values of 0.173, 0.166, and 0.154, respectively.

#### 3.7.3. Radius of Gyration (Rg) and Solvent Accessible Surface Area (SASA)

**Figure 7:**
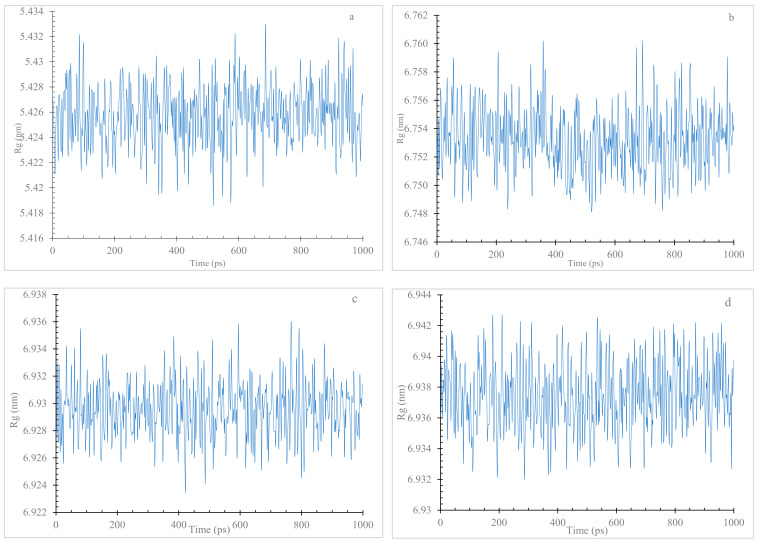
The Rg of C atoms in native and mutant α-Syn protein types. The wildtype (**a**), the V82A (**b**), the K80E (**c**), and the E46K (**d**).

Understanding the structural consequences of SNCA mutations is critical for unraveling the molecular mechanisms of Parkinson’s disease. The MD simulation results (Figure 7a–c) highlight the significance of V82A, K80E, and E46K mutations in promoting protein flexibility and altering the native conformation. The Rg values of the wildtype α Syn (between 5.42 nm and 5.43 nm) remained relatively stable throughout the simulation, indicating a compact and well-defined structure. In contrast, all three mutant variants (V82A, K80E, and E46K) consistently displayed higher Rg values (6.75 nm and 6.76 nm, 6.92 nm and 6.94 nm, 6.93 nm, and 6.94 nm, respectively).

**Figure 8:**
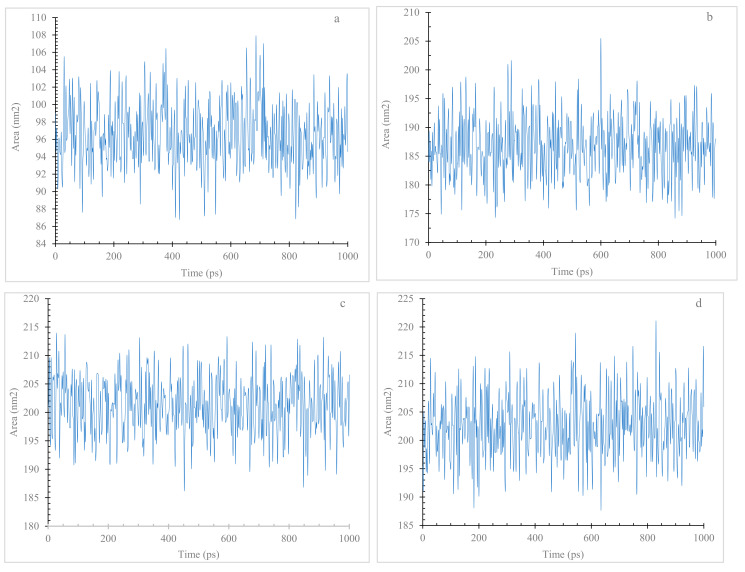
Solvent accessible surface area (SASA) of wildtype and mutant forms of α-Syn protein. Wildtype (**a**), V82A (**b**), K80E (**c**), and E46K (**d**) mutants.

Through SASA analysis, the present finding provides insights into the structural impacts of mutations (V82A, K80E, and E46K) within the SNCA gene. The observed elevation in SASA values suggests a potential link between these mutations and altered structural dynamics (Figure 8a–d), contributing to our understanding of PD pathology. Comparative analysis of SASA values unveiled distinct trends for the mutant variants in contrast to the wildtype α-Syn. The V82A, K80E, and E46K mutants consistently exhibited higher SASA values throughout the simulations.

## 4. Discussion

Parkinson’s disease (PD) is very common neurodegenerative disease affecting movement [31]. The risk factor of PD are age, male gender, and some environmental risk factors [31]. The cause of PD is still to be clarified but different genes were reported [31]. The α-Syn is an abundant neuronal protein associated with the pathology of PD and other neurodegenerative disorders called synucleinopathies. The synucleinopathies are grouped into the Lewy body disease and multiple system atrophy (MSA) [32]. The Lewy body disease is a neurodegenerative disease in which the aggregates of α-Syn accumulated in the perikarya and neurites of the neurons (Lewy bodies and Lewy neurites) [33]. While the MSA is a neurodegenerative disorder caused by accumulation of the misfolded α-Syn in oligodendrocytes, where it builds the glial cytoplasmic inclusions (GCIs) [34]. α-Syn is a 14 kDA protein encoded by the SNCA gene. It is found in Lewy bodies, oligomers, fibrils of α-Syn, and mutations SNCA gene in N-terminal region of α-Syn are associated with the risk of PD [35]. It has been reported that the mutated α-Syn form oligomerization and fibrillation faster than the wildtype form of α-Syn, and that the treatment with dopamine promotes the polymerization rate of mutated and wildtype α-Syn [35,36]. The α-Syn monomers accumulation initiates the deposition of aggregates that is a characteristic of synucleinopathies [35].

It has been reported that the aggregation of α-Syn relies on the pH, post-translational modifications (PTM) and its concentration [37]. The α-syn concentration promotes its associations, or results in α-syn precipitation when it is beyond solubility limit [37]. The PTMs (e.g., phosphorylation, ubiquitination, and truncation [8]), hampers the α-syn aggregation via influencing α-syn topology and its surface charge [38]. In addition, low pH enhances aggregation of α-syn by increasing exposure of hydrophobic core [38]. The development of α-syn fibrils is suppressed at pH close to neutral pH, whereas α-syn fibrils progression occurs at slightly acidic pH values (5.8) [38]. The pH influences the intramolecular folding and protein-protein interactions of α-syn [37]. Moreover, metal ions, for example copper, zinc, iron, and calcium, have also been demonstrated to influence the rate of α-syn aggregation [37]. Certain natural polysaccharides were reported to confer neuroprotective properties in various neurodegeneration models [39]. For instance, a green algae polysaccharide was demonstrated to exert anti-Parkinson’s disease properties via suppression of α-Syn fibrillation. It has been reported these natural polysaccharides enhance the cellular autophagy and thereby suppress the α-Syn fibrillation [39].

Nucleotide variations influence the physiological processes and contributing to various pathological conditions [40,41,42,43,44,45,46,47,48]. The pathogenic mutations can adversely affect protein function, protein-protein interactions (PPI), charge or spatial structure [49,50]. Mutation can cause loss of hydrophobicity, instability, misfolding, and aberrant PPI [49,50,51]. Mutations (e.g., G51D and A30G) in the SNCA gene were associated with PD [52,53]. The objective of this investigation was to examine the nsSNP in the SNCA gene utilizing various in silico methodologies such as SIFT, PolyPhen2, SNAP2, SNP&GO, PhD-SNP, I-Mutant, Mu-Pro, ConSurf, HOPE, Mut-pred, and MD simulation. This study’s methodology primarily centers on examining the relationship between molecular-level variations and their impact on the protein. Employing a diverse range of tools for a singular objective engenders a sense of assurance in the outcomes, given that each software or tool operates on a distinct algorithm. The outcomes derived from the agreement among all the utilized tools and web servers possess the utmost precision [54]. The functional consequences of nsS (Table 2). NPs on the SNCA gene were evaluated through the utilization of four computational prediction tools, namely SIFT, PolyPhen2, SNAP2, and Panther (Table 1). Additionally, two other tools, PhD-SNP and SNP&GO, were employed to predict the plausible correlation between nsSNPs and disease. This revealed 20 nsSNPs that are potentially “high risk” and deleterious; these twenty nsSNPs were employed in additional prognostic evaluations. This study evaluated the protein stability of 20 nsSNPs with high-risk potential using I-mutation2 and MuPro. Our results indicated that four of the nsSNPs led to a reduction in protein stability (Table 3). The results of the phylogenetic analysis revealed that the nsSNPs were located in regions exhibiting elevated conservation scores (Figure 2). The protein’s function may be significantly affected by the variations in these regions (Table 4) [49].

The molecular mechanism of pathogenic analysis utilizing MutPred2 has indicated that the V82A mutation is found in NAC region [6] and results in the absence of Pyrrolidone carboxylic acid at position Q79 of the α-Syn protein (Table 4). V82A α-Syn mutant is smaller than wildtype α-Syn. The V82A α-Syn mutant is located near a highly conserved position and mutant residue’s smaller size may lead to loss of interactions (Table 5).

According to the MutPred2 analysis, the K80E mutation present in NAC region [6], and leads to destabilization of the protein due to the loss of methylation and ubiquitination [55] at the K80 site (Table 4). Methylation of protein has important roles in modulation of the function of protein and regulation of physiological process [56]. In addition, in the K80E the K (lysine) is a positively charged amino acid and is replaced by the E (glutamic acid) which is a negatively charged amino acid. This may have a profound effect on the function and structure of α-Syn. The K80E α-Syn mutant is smaller than the wildtype and mutant residue’s smaller size may lead to loss of interactions (Table 5).

Furthermore, the E46K mutation found in the N-terminal [6], and results in modified transmembrane properties acquisition of methylation at E46, and a modified disordered interface in the α-Syn protein, potentially leading to changes in the protein’s inherent functions [57]. The E46K mutant is bigger than the wildtype and may lead to the formation of bumps (Table 5). In addition, in the E46K mutant, E (glutamic acid) which is a negatively charged amino acid is substituted with K (lysine) which is a positively charged amino acid, which may have a serious effect on α-Syn structure and function. The E46K mutation results in the formation of a distinct fibril structure in α-Syn that leads to development of PD [58]. This result is in agreement with a study by Zhao et al. [58]. It has been reported that α-Syn fibrillation induced by E46K mutation (and other mutations, e.g., A30P, A53T and E35K, and E57K) can be inhibited by polysaccharides isolated from *Chlamydomonas reinhardtii* called Cr-SPS, and that Cr-SPS promotes the solubility of α-Syn [39].

In addition, our results indicated that the A18G mutation (Table 2) could occur in α-Syn protein. Change the alanine a hydrophobic amino acid to glycine, hydrophilic amino acid [59] may affect protein structure and function [49], however, our MutPred result showed that it would not influence α-Syn (Table 4). This result may be investigated in future studies. Furthermore, our analysis could reproduce the mutation A30P (Table 2), which is already mentioned to be associated with PD [17,18]. The fibrillation of the A30P α-Syn mutant was shown to be inhibited by minimalistic compound, ZPDm [60]. Our MutPred result showed that the A30P may not affect α-Syn protein (Table 4). However, this mutation may be examined in future studies.

We also found the mutation V52A (Table 2) which between the reported mutations A53T, G51D [17,18]. Since V52A is close to reported mutations A53T, G51D, it is plausible that V52A can influence α-Syn structure and function. However, this should await future investigations.

**Figure 9:**
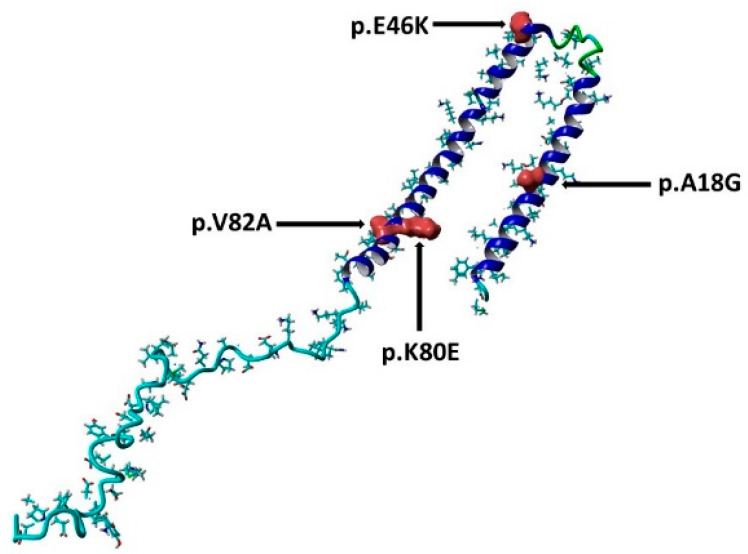
The three-dimensional structure of the α-syn protein (PDB ID:1XQ8), the sites of the mutations are shown in surface presentation and red color. This figure is prepared using YASARA view.

In order to assess the protein dynamics of wildtype and α-Syn mutant forms, a MD simulation was conducted. Specifically, various parameters, including temperature, pressure density, RMSD, RMSF, Rg, and SASA, (Figure 3, Figure 4, Figure 5, Figure 6, Figure 7 and Figure 8) were compared and analyzed. The comparisons were conducted in order to analyze the conformational characteristics of each protein structure with respect to its stability. Following the minimization process, it was observed that there was a slight variation in the temperature (Figure 3), pressure (Figure 4), and density (Figure 5) between the native and mutant conformations over the course of time. The RMSD plots of the backbone atoms indicated that the stability of the wildtype structure was significantly greater than that of the α-Syn mutant forms (Figure 6). The RMSF plots indicate that both the native and mutant structures exhibited the highest level of flexibility (Figure 6). The RMSF computed across the trajectories across the residual components indicated that the atomic fluctuation in the structures was more pronounced in the initial and final quartiles. The radius of gyration (Figure 7) and solvent accessibility of surface area (Figure 8) analysis were utilized to investigate the impact of the unfolding of the mutant protein structure on the solubility of its surface area and the resulting reduction in protein activity. A correlation has been established between the Rg value and the stability of a system, whereby a higher Rg value is indicative of lower stability [61]. This result indicates that the native structure exhibits a narrower range of Rg in comparison to the mutants (Figure 7). This result suggests that these mutations (p.V82A, p.K80E, and p.E46K) (Figure 9) introduce structural perturbations, potentially affecting the stability and function of the protein. Moreover, the SASA results for α Syn mutants were significantly elevated compared to the wildtype α Syn. These findings suggest a plausible increase in solvent exposure due to mutations, potentially influencing protein–protein and protein–environment interactions. This result provides evidence that the mutations V82A, K80E, and E46K (Figure 7b–d and Figure 8a–c) have a destabilizing effect on protein, resulting in reduced protein functionality. Although the graphs of the radii of gyration appear to be in equilibrium (Figure 7), further studies with longer simulations will be needed to confirm these results. The study presented a robust platform for conducting virtual screening of SNCA gene through a systematic and all-encompassing approach. Additionally, it uncovered the molecular basis for recognizing alterations in the structure, function, stability, and other characteristics of α-Syn. Limitations of the present study include that the effects of the pH, the α-Syn protein concentration, metal ions, and natural compounds that interact with the residues crucial for the protein aggregation (e.g., natural polysaccharides) on the α-Syn mutants have not been investigated. Future studies examining the effects of the above-mentioned factors on these mutations are recommended. In addition, further studies investigate the effect of these mutations of α-Syn function, PPI, and well-designed case–control studies [62,63,64,65] are warranted to verify these results.

## 5. Conclusions

This study investigated the potential association between nsSNPs in the SNCA gene and PD using a variety of computational techniques, including SIFT, PolyPhen2, Mut-pred, SNAP2, PANTHER, PhD-SNP, SNP&Go, MUpro, Cosurf, and I-Mut. The results suggest that the stability and functionality of the α-Syn protein encoded by the SNCA gene may be affected by three potential pathogenic mutations, p.V82A, p.K80E, and E46K. The development of Parkinson’s disease may be influenced by these changes in protein properties. Once these findings have been verified, they can be used to assess the risk level and select the best course of treatment (e.g., with natural polysaccharides) or preventative measures for genetic testing of Parkinson’s disease and other synucleinopathies.

## Figures and Tables

**Table 1:** SIFT, PolyPhen, SNAP2, and PANTHER predictions for the impact of amino acid substitution on the SNCA gene.

					SIFT		Polyphen2		SNAP2		PANTHER	
Variant ID	Alleles	MAF	Transcript ID	Mutation	Sift Score	Prediction	Polyphen Score	Prediction	Prediction	Accuracy %	pdel	Prediction
rs1319839593	A|G	G = 0.000008/2	ENST00000673718.1	V82A	0	deleterious	0.999	probably damaging	effect	0.8	456	probably damaging
rs1261243630	T|C	C = 0.000004/1	ENST00000673718.1	K80E	0	deleterious	0.96	probably damaging	effect	0.75	456	probably damaging
rs1239518140	A|G	G = 0.000004/1	ENST00000673718.1	V52A	0	deleterious	1	probably damaging	effect	0.8	456	probably damaging
rs104893875	C|T	T = 0./0	ENST00000673718.1	E46K	0	deleterious	0.959	probably damaging	effect	0.66	455	probably damaging
rs750512067	C|T	T = 0.000008/2	ENST00000673718.1	G41D	0	deleterious	0.998	probably damaging	effect	0.95	456	probably damaging
rs750745088	C|A	T = 0.000008/1	ENST00000673718.1	V37F	0	deleterious	0.949	possibly damaging	effect	0.8	456	probably damaging
rs1342686707	C|G	G = 0.000004/1	ENST00000673718.1	G36R	0	deleterious	1	probably damaging	effect	0.8	456	probably damaging
rs1342686707	C|T	G = 0.000004/1	ENST00000673718.1	G36S	0	deleterious	0.999	probably damaging	effect	0.71	456	probably damaging
rs1330229174	T|C	C = 0.000004/1	ENST00000673718.1	K34E	0	deleterious	0.96	probably damaging	effect	0.66	456	probably damaging
rs104893878	C|G	N/A	ENST00000673718.1	A30P	0	deleterious	0.996	probably damaging	effect	0.8	456	probably damaging
rs753674628	C|T	T = 0.000008/2	ENST00000673718.1	V26M	0	deleterious	0.996	probably damaging	effect	0.53	456	probably damaging
rs1433622151	C|T	T = 0.000004/1	ENST00000673718.1	G25S	0	deleterious	0.999	probably damaging	effect	0.75	456	probably damaging
rs1273319141	G|A	A = 0.000004/1	ENST00000673718.1	T22I	0	deleterious	0.999	probably damaging	effect	0.8	456	probably damaging
rs778867145	T|C	C = 0.000016/2	ENST00000673718.1	E20G	0	deleterious	1	probably damaging	effect	0.85	456	probably damaging
rs752472160	G|C	C = 0.003975/467	ENST00000673718.1	A18G	0	deleterious	0.998	probably damaging	effect	0.75	456	probably damaging
rs1289802008	A|G	G = 0.000004/1	ENST00000673718.1	V16A	0	deleterious	1	probably damaging	effect	0.63	456	probably damaging
rs1739238968	A|G	G = 0.000007/1	ENST00000673718.1	V15A	0	deleterious	1	probably damaging	effect	0.85	456	probably damaging
rs1219278381	C|A	A = 0.000004/1	ENST00000673718.1	A11S	0	deleterious	0.887	possibly damaging	effect	0.75	456	probably damaging
rs1188720061	T|G	G = 0.000004/1	ENST00000673718.1	K6N	0	deleterious	1	possibly damaging	effect	0.75	456	probably damaging
rs1168809349	A|T	T = 0.000007/1	ENST00000673718.1	V3E	0	deleterious	0.972	possibly damaging	effect	0.71	456	probably damaging

**Table 2:** Analysis of Disease-Associated nsSNPs on SNCA gene.

			PhD-SNP		SNP&GO	
Variant ID	Alleles	Mutation	Score	Prediction	Score	Prediction
rs1319839593	A|G	V82A	1	Disease	5	Disease
rs1261243630	T|C	K80E	3	Disease	8	Disease
rs1239518140	A|G	V52A	3	Neutral	6	Disease
rs104893875	C|T	E46K	0	Disease	9	Disease
rs750512067	C|T	G41D	5	Neutral	9	Disease
rs750745088	C|A	V37F	2	Neutral	9	Disease
rs1342686707	C|G	G36R	3	Neutral	9	Disease
rs1342686707	C|T	G36S	7	Neutral	9	Disease
rs1330229174	T|C	K34E	1	Neutral	9	Disease
rs104893878	C|G	A30P	1	Neutral	10	Disease
rs753674628	C|T	V26M	3	Neutral	8	Disease
rs1433622151	C|T	G25S	1	Neutral	9	Disease
rs1273319141	G|A	T22I	3	Neutral	9	Disease
rs778867145	T|C	E20G	1	Neutral	8	Disease
rs752472160	G|C	A18G	0	Disease	8	Disease
rs1289802008	A|G	V16A	5	Neutral	5	Disease
rs1739238968	A|G	V15A	1	Neutral	5	Disease
rs1219278381	C|A	A11S	5	Neutral	9	Disease
rs1188720061	T|G	K6N	4	Neutral	8	Disease
rs1168809349	A|T	V3E	4	Neutral	8	Disease

**Table 3:** Effects of SNPs on the α-Syn protein stability, increasing or decreasing.

Variant ID	Allele	Mutation	I-Mutant	MuPro
rs1319839593	A|G	V82A	Decrease	Decrease
rs1261243630	T|C	K80E	Decrease	Decrease
rs104893875	C|T	E46K	Decrease	Decrease
rs752472160	G|C	A18G	Decrease	Decrease

**Table 4:** Investigation of the molecular mechanisms underlying pathogenicity.

				MutPred	
Variant ID	Allele	Mutation	Score	Effect	Function Affected
rs1319839593	A|G	V82A	0.637	−	Loss of Pyrrolidone carboxylic acid at Q79
rs1261243630	T|C	K80E	0.701	−	Loss of Methylation at K80; Loss of Ubiquitylation at K80
rs104893875	C|T	E46K	0.646	+	Gain of Methylation at E46; Altered Disordered interface; Altered Transmembrane protein
rs752472160	G|C	A18G	0.433	no results	No effects produced

**Table 5:** Summary of the results of HOPE analysis for the three different amino acid mutations of SNCA gene.

Mutation	V82A	K80E	E46K
AA Properties			
Size	Mutant is smaller than wild type	Mutant is smaller than wild type	Mutant is bigger than wild type
Charge	No charge change	Wildtype charge: POSITIVE	Wildtype charge: NEGATIVE
		Mutant charge: NEGATIVE	Mutant charge: POSITIVE
Structure		Preferred secondary structure for wild type, destabilized by mutant	
		secondary structure preference for mutant	
Conservation			
Conservation	Mutant located near a highly conserved position	Only this residue type at position, mutation possibly damaging	Mutant located near a highly conserved position
		Mutation may be damaging	Mutation may be damaging
Conclusion			
AA Properties	Mutant residue’s smaller size may lead to loss of interactions	Charge difference between wild type and mutant could cause repulsion	Charge difference between wild type and mutant could cause repulsion
		Mutant residue’s smaller size may lead to loss of interactions	Mutant residue’s bigger size may lead to bumps

HOPE analysis results for the three different amino acid mutations, including information about size, charge, structural preferences, and conservation. AA: amino acid.

## Data Availability

The authors confirm that the data supporting the results of this study are available within the article.
